# Effect of cell wall polysaccharides on the peelability in table grape berries

**DOI:** 10.3389/fpls.2025.1605812

**Published:** 2025-05-21

**Authors:** Boxiang Zhao, Junfei Bi, Haonan Wang, Mingyuan Wang, Wei Ji

**Affiliations:** ^1^ 1Institute of Horticulture Science and Engineering, Huaqiao University, Xiamen, China; ^2^ College of Horticulture, Shanxi Agricultural University, Jinzhong, China

**Keywords:** table grape, cell wall polysaccharides, peelability, skin-pulp adherence, grape maturity

## Abstract

Grape peelability varieties meet the demand for convenience and hygiene and are popular Grape varieties with easy peelability meet consumer demands for convenience and hygiene, making them increasingly popular. Differences in grape peelability are likely associated with variations in cell wall polysaccharide composition in the pulp and skin. Twelve table grape varieties (‘Zaoheibao’, ‘Qiuhongbao’, ‘Summer Black’, ‘Black Balado’, ‘Jinghongbao’, ‘Lihongbao’, ‘Flame Seedless’, ‘Crimson Seedless’, ‘Wanheibao’, ‘Wuhecuibao’, ‘Thompson Seedless’ and ‘Hutai No. 8’) were selected to investigate skin-pulp adherence, skin cell morphology, and cell wall polysaccharide content during fruit development. The role of cell wall polysaccharides in peelability was evaluated by assessing skin-pulp adherencce, skin cell morphology, cell wall polysaccharide content, and activities of related degrading enzymes across developmental stages of different grape varieties. Results showed that skin-pulp adherencce decreased by 6.4%~52.4% during fruit development, with significant varietal differences. ‘Black Balado’ exhibited the highest adhesion, while ‘Flame Seedless’ had the lowest. Cluster analysis grouped ten Eurasian grape varieties into two categories. The first group, which was easier to skin, included ‘Flame Seedless’, ‘Thompson Seedless’, ‘Wuhecuibao’, ‘Zaoheibao’, ‘Wanheibao’, ‘Jinghongbao’, ‘Lihongbao’, ‘Qiuhongbao’ and ‘Wuhebai’. The second group, characterized by poor peelability, included ‘Black Balado’. Anatomical observations revealed that as fruit development progressed, intercellular adhesion weakened and pulp cell separation became more pronounced. As fruit developed, cell wall polysaccharide content (cell wall material, cellulose, hemicellulose, protopectin, chelator-soluble pectin, water soluble pectin) decreased, while the activities of related degrading enzymes (cellulase, β-glucosidase, xylanase, xyloglucan endotransglycosylase, β-mannanase, polygalacturonase, pectate lyase, pectin methyl esterase, β-galactosidase, α-L-arabinofuranosidase) gradually increased. Specifically, the cell wall material content of the cell walls decreased by 30.3% to 64.8% in the pulp and by 23.9% to 51.4% in the pericarp across different varieties. protopectin and chelator-soluble pectin showed the most significant declines. In ‘Flame Seedless’ peel, protopectin content decreased by 97.1%, from 2067 µg•g^–1^ FW at the expansion stage to 60 μg•g^–1^ FW at maturity, and by 93.9% in the pulp. Chelator-soluble pectin content decreased by 87.8% to 97.7% in the peel and by 73.7% to 94.6% in the pulp, depending on the variety. The activities of cellulase and β-glucosidase showed relatively moderate changes during fruit development. From the expansion to the ripening stage, xylanase activity increased by 0.37-2.55 times in the peel and 0.01-1.84 times in the pulp. Similarly, xyloglucan endotransglycosylase activity rose by 0.38-2.37 times in the peel and 0.42-2.33 times in the pulp, while polygalacturonase activity increased by 0.21-2.85 times in the peel and 0.58-2.43 times in the pulp. Pectate lyase activity increased from 16% to 43% before the veraison stage and from 1% to 11% afterward. During both the expansion-to-verasion and verasion-to-ripening stages, pectin methyl esterase activity increased by 0.69-1.07-fold in the peel and 0.29-1.53-fold in the pulp, while β-galactosidase activity increased by 0.21-0.55-fold in the peel and 0.05-1.02-fold in the pulp. α-L-arabinofuranosidase activity increased by 1% to 341% in the peel and by 85% to 365% in the pulp. This study found that the peelability of table grapes gradually decreased during fruit ripening and varied significantly among different varieties. Further analysis indicated that peelability was negatively correlated with cell wall polysaccharide content and positively correlated with the activity of related cell wall-degrading enzymes. This study provides a theoretical framework for understanding the physiological mechanisms underlying grape peelability.

## 1 Introduction

Grapes (*Vitis* spp.) hold a significant place in the global fruit industry and have long been ranked among the world’s top six fruits due to their unique growth habits and rich nutritional value (Dong et al., 2025). The peelability of high-quality table grape varieties meets consumer demand for convenience and serves as a key indicator of fruit quality (Xu et al., 2023; Simos et al., 2018). The formation of fruit peelability is a complex process influenced by multiple factors, including cultivar characteristics, pericarp structure, cell wall polysaccharides, and related degrading enzymes (Tao et al., 2024). Yu et al. (2021) reported that among four citrus types, pummelo exhibited the highest skin-pulp adherence, while mandarin showed the lowest. Based on skin-pulp adherence, 80 citrus varieties were classified into 42 highly peelablility, 30 moderately peelablility, and 8 difficult-to-peel categories. Peel structure was closely associated with peelability. Easy-to-peel citrus varieties typically have loose rinds largely separated from the pulp, while difficult-to-peel varieties have rinds that are tightly attached (Goldenberg et al., 2018). Research on *Actinidia eriantha* (Tao et al., 2024) also suggests that pericarp structure may influence fruit peelability. In addition, peelability is genetically regulated by multiple genes and is also influenced by year effects and ripening time (Nishio et al., 2022). Moreover, peelability is associated with the composition of cell wall polysaccharides and the enzymes related to their degradation (Qu et al., 2022; Zhang et al., 2021).

Cell wall polysaccharides, such as pectin, hemicellulose, and cellulose, are essential structural components that play a critical role in maintaining fruit cell integrity (Huang et al., 2022). Structural changes and degradation of cell wall components during fruit ripening and postharvest storage are major contributors to fruit quality deterioration (Ren et al., 2020; Zhao et al., 2019). Analysis of citrus fruits revealed significant differences in cell wall polysaccharide content among ‘Hongmeiren’, ‘Satsuma’, and ‘Nanfeng’ tangerines (Yao et al., 2023). Easy-to-peel varieties exhibited lower total cell wall biomass and lower levels of hemicellulose, cellulose, and pectin compared to hard-to-peel varieties (Zhang et al., 2021). Atkinson et al. (2009) found that the distribution and composition of pectin in peel and pulp tissues differed between highly and poorly peelable varieties, which may largely account for the variation in peelability. During fruit ripening, enzymes including polygalacturonase, pectin methylesterase, pectin lyase, β-galactosidase, and cellulase participate in the degradation of cell wall polysaccharides (Yu et al., 2023). β-galactosidase contributes to the degradation of pectin and hemicellulose, leading to cell wall loosening and subsequent changes in fruit texture (Feng et al., 2019). In grapes, fruit deterioration can be effectively delayed by suppressing the activity and gene expression of cell wall-modifying enzymes such as polygalacturonase, cellulase, pectin methylesterase, and β-galactosidase (Fan et al., 2019; Shi et al., 2019). Variations in pectin methyl esterification, galactose loss, and the activities of polygalacturonase and xyloglucan endotransglucosylase/hydrolase in peel and pulp tissues are key factors influencing the peelability of *A. eriantha* (Prakash et al., 2017). Currently, the mechanisms by which grape cell wall polysaccharides influence peelability remain poorly understood.

In this study, twelve table grape varieties will be used to investigate the textural properties, cellular morphology, and metabolic changes of the skin and pulp during fruit development. Key parameters include skin–pulp adhesion, skin cell morphology, cell wall polysaccharide content, and the activity of enzymes involved in polysaccharide degradation. The aim is to elucidate the physiological basis underlying grape peelability traits and provide theoretical support for the improvement of peelability in table grapes.

## 2 Materials and methods

### 2.1 Materials and reagents

From July to October 2022, grape plants with vigorous growth and healthy appearance were selected, and disease-free berries were randomly harvested during the expanding (July-August), veraison (August-September), and rpening (September-October) stages. Skin-pulp adherence was measured in the laboratory, and paraffin sections were prepared for microscopic observation. The harvested grape skins and flesh tissues were rapidly frozen at -80 °C for subsequent analysis of polysaccharide content and the activity of enzymes related to polysaccharide degradation. The 12 table grape varieties selected for this study included ‘Zaoheibao’ (*V vinifera*), ‘Qiuhongbao’ (*V vinifera*), ‘Summer Black’ (*V vinifera*×*V labrusca*), ‘Black Balado’ (*V vinifera*), ‘Jinghongbao’ (*V vinifera*), ‘Lihongbao’ (*V vinifera*), ‘Flame Seedless’ (*V vinifera*), ‘Crimson Seedless’ (*V vinifera*), ‘Wanheibao’ (*V vinifera*), ‘Wuhecuibao’ (*V vinifera*), ‘Thompson Seedless’ (*V vinifera*), and ‘Hutai No.8’ (*V vinifera*×*V labrusca*) All of these berries were collected from the Grape Germplasm Resources Nursery of the Horticultural Station of Shanxi Agricultural University(37°23′N 112°32′E, altitude 830 m).

### 2.2 Determination of skin-pulp adherence

The tops of individual grape berries were removed, and cylindrical sections (10 mm thick), including both skin and pulp, were taken from the equatorial region. The skin was gently scraped with a razor blade and folded outward by approximately 5 mm. The skin–pulp adherence force was measured using a digital dynamometer (ZMF-3, Weidu, Zhejiang) equipped with a jig (HJJ-001, Edelberg, Zhejiang). The outer rind was clamped using the jig, and the pulp was pulled vertically downward with forceps until complete separation occurred. The maximum value recorded by the dynamometer was taken as the skin-pulp adherence force (N), replicated ten times in total.

### 2.3 Morphological observation of fruit epidermal cells

The vertical pericarp was cut from the equatorial region of the fruit into 0.5 cm × 0.5 cm × 0.5 cm pericarp squares with pulp, and was fixed using FAA (containing 90 mL 70% alcohol, 5 mL glacial acetic acid, and 5 mL 38% formaldehyde). This was followed by paraffin sectioning, ethanol gradient dehydration, xylene transparency, paraffin embedding, safranin O-fast green staining, and sealing with Canada gum. The sections were then placed under an ordinary light microscope to select the sections for observation and photographed using an Olympus DP71 (Japan) microimaging system.

### 2.4 Determination of cellulose, hemicellulose, pectin content and related enzyme activities

The cell wall content was determined according to the method described by Zhang et al. (2021). Approximately 0.3 g of peel and pulp tissue was weighed, ground, mixed with 1 mL of 80% ethanol, and heated in a water bath at 95 °C for 20 minutes. After cooling to room temperature, the mixture was centrifuged at 4,000 × g for 10 minutes at 25 °C, and the supernatant was discarded. The pellet was washed once with 1.5 mL of 80% ethanol and acetone, then vortexed for 2 minutes. It was then soaked in 1 mL of 90% DMSO solution for 15 hours to remove starch. After soaking, the sample was centrifuged at 4,000 × g for 10 minutes at 25 °C, and the supernatant was discarded. The resulting pellet was dried at 45 °C to a constant weight to obtain the cell wall material. Weighing 0.1 g of dried cell wall material of cell wall, the cellulose and hemicellulose contents were determined by 72% concentrated sulfuric acid hydrolysis and 2 mol/L hydrochloric acid hydrolysis (Zhao et al., 2021), respectively, in mg·g^-1^ FW. The extraction of protopectin, chelator-soluble pectin, and water-soluble pectin was carried out by the method of Qi et al. (2015), and the content was determined by carbazole-sulfuric acid colorimetric method in μg·g^-1^ FW. Cellulase, xylanase and polygalacturonase activities were determined by DNS colorimetric method (Lo’ay and EI-Khateed, 2018). Salicin hydrolysis method was used for the determination of β-glucosidase activity (Su et al., 2017). Pectin lyase enzyme activity was determined with reference to the method of Sathiyaraj et al. (2011). Determination of xyloglucan endotransglycosylase and pectin methylesterase activities was carried out by the method of Ye (2014). The DNS colorimetric method (Tian et al., 2020) was used for the determination of β-mannanase activity. The ONPG colorimetric method (Zhang et al., 2021) was used for the determination of β-galactosidase activity. Determination of α-L-arabinofuranosidase activity was carried out by p-nitrophenyl arabinofuranoside method (Li et al., 2023). The results were repeated three times.

### 2.5 Data processing

Microsoft Excel 2016 was used for data organization; SPSS 19.0 was used for significance analysis, Pearson’s correlation analysis and cluster analysis of the experimental data; Duncan’s multiple comparison Method was used to determine significant differences (*p*<0.05). XLSTAT, The Unscrambler X 10.4 was used for partial least squares regression analysis; OriginPro 2021, Adobe Photoshop CC 2018 was used for plotting.

## 3 Results and analysis

### 3.1 Changes of skin-pulp adherence of different varieties of grapes in the process of fruit development

**Figure 1:**
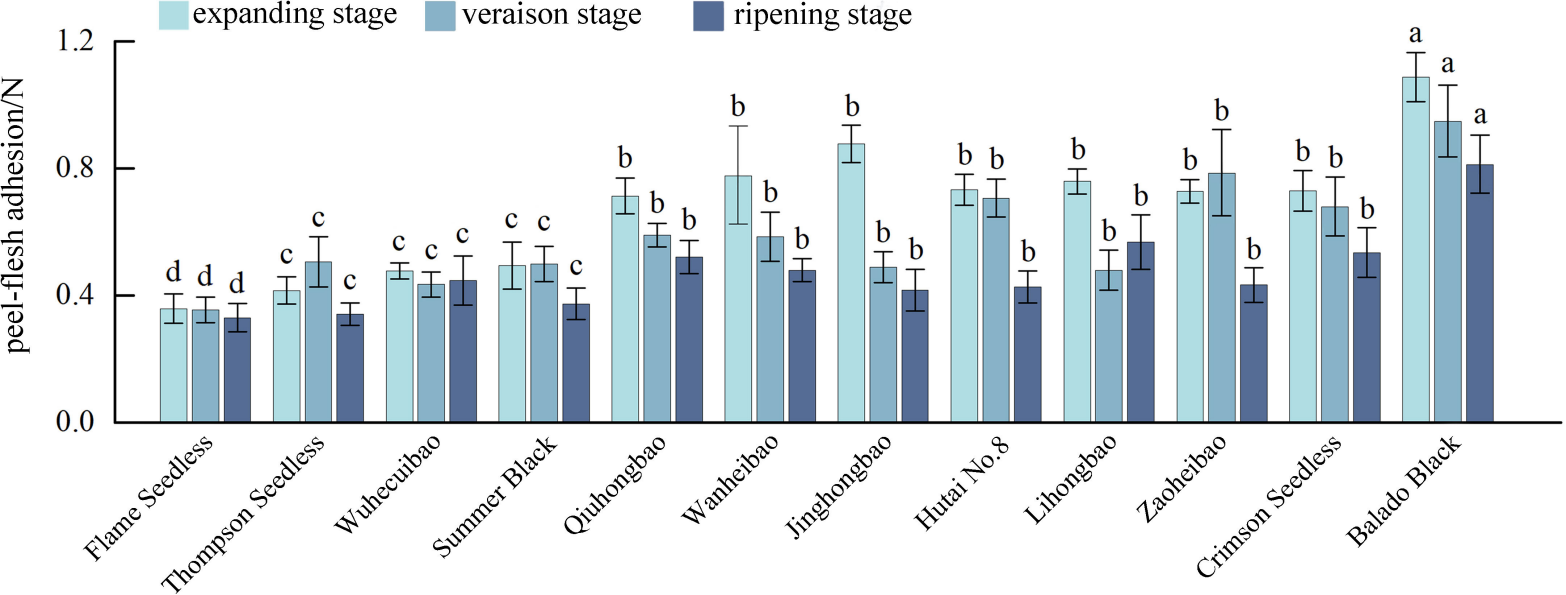
Peel-flesh adherence during the development of different varieties of grapes. Different lowercase letters in the figure indicate significant differences between different varieties (P ≤ 0.05).

**Figure 2:**
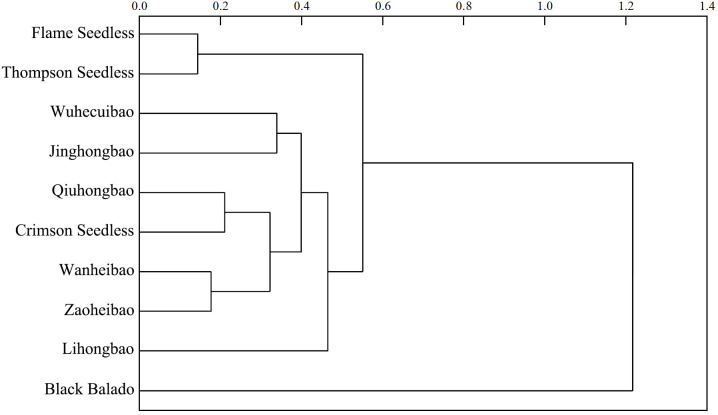
Cluster analysis of peelability of different varieties of grapes based on the peel-flesh adhesions at ripening stage.

In the process of fruit development, the skin-pulp adherence of ‘Flame Seedless’, ‘Qiuhongbao’, ‘Wanheibao’, ‘Jinghongbao’, ‘Hutai No.8’, ‘Crimson Seedless’, and ‘Black Balado’ showed a decreasing trend. ‘Thompson Seedless’, ‘Summer Black’, and ‘Zaoheibao’ showed an increasing-then-decreasing trend, while ‘Wuhecuibao’ and ‘Lihongbao’ showed a decreasing-then-increasing pattern (**Figure 1**). However, for the five varieties, skin-pulp adherence at maturity was lower than during the expansion stage. The reduction in skin-pulp adherence among different grape varieties ranged from 6.4% to 52.4%, suggesting that peelability may develop progressively during fruit maturation. Statistical analysis classified grape skin-pulp adherence into four significantly distinct levels. Among them, ‘Flame Seedless’ exhibited the lowest skin-pulp adherence. This was followed by ‘Thompson Seedless’, ‘Wuhecuibao’, and ‘Summer Black’, all significantly lower than ‘Qiuhongbao’, ‘Wanheibao’, ‘Jinghongbao’, ‘Hutai No.8’, ‘Lihongbao’, ‘Zaoheibao’, and ‘Crimson Seedless’. During the expansion, veraison, and ripening stages, ‘Black Balado’ had significantly higher skin-pulp adherence than other varieties. Cluster analysis of skin-pulp adherence during ripening divided the ten Eurasian grape varieties into two categories: The first group included easy-peel varieties, including ‘Flame Seedless’, ‘Thompson Seedless’, ‘Wuhecuibao’, ‘Wanheibao’, ‘Zaoheibao’, ‘Jinghongbao’, ‘Lihongbao’, ‘Qiuhongbao’, and ‘Crimson Seedless’. The second group comprised hard-peel varieties, such as ‘Black Balado’ (**Figure 2**).

### 3.2 Changes in the morphology of pericarp cells of different varieties of grapes in the process of fruit development

**Figure 3:**
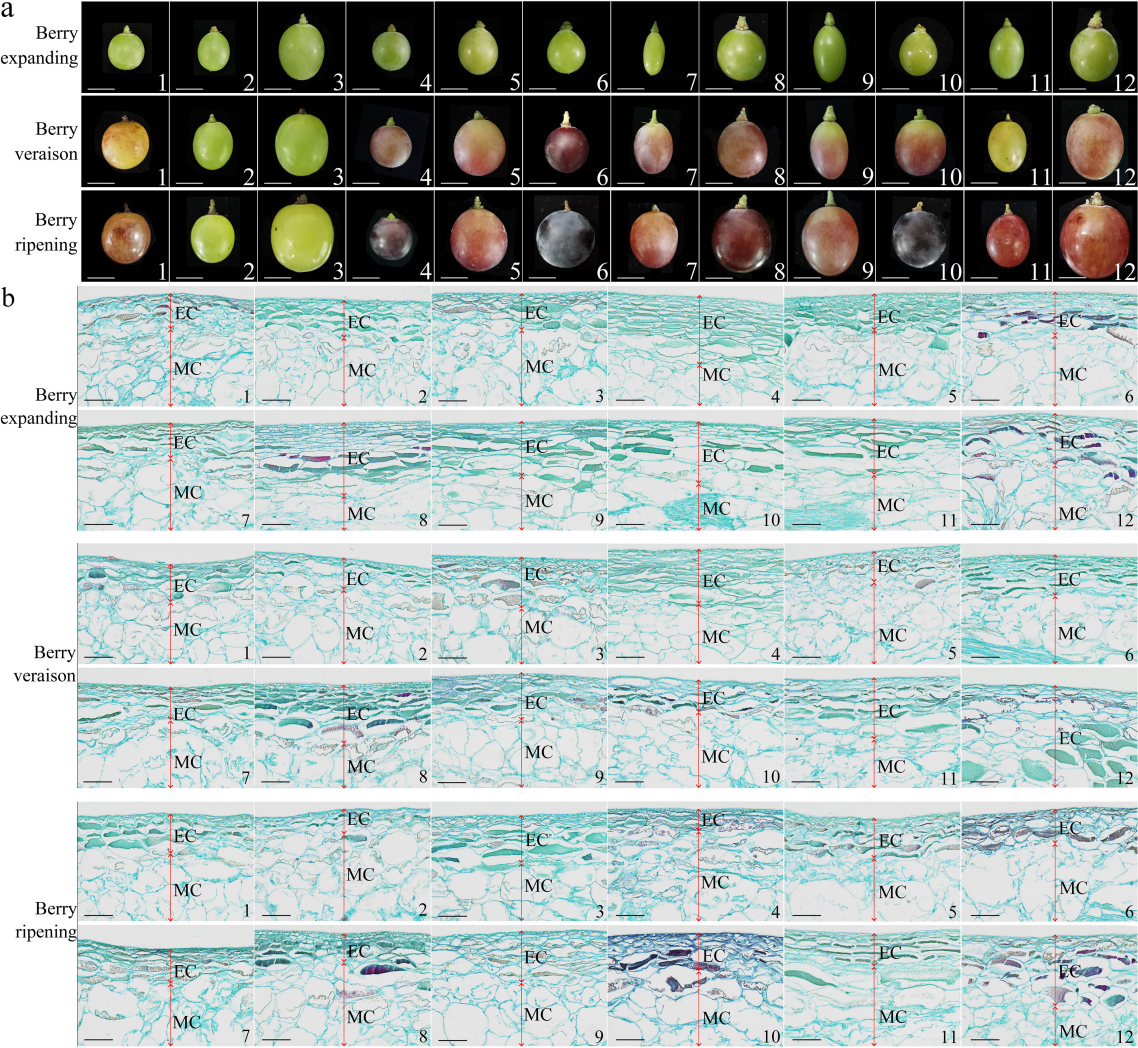
Differences in appearance and anatomical structure during the development of different varieties of grapes. **(a)**: The differences in appearance during the development of different varieties of grapes; **(b)**: Anatomical structural differences during the development of different varieties of grapes. 1, 2, 3, 4, 5, 6, 7, 8, 9, 10, 11, 12 represent: ‘Flame Seedless’, ‘Thompson Seedless’, ‘Wuhecuibao’, ‘Summer Black’, ‘Qiuhongbao’, ‘Wanheibao’, ‘Jinghongbao’, ‘Hutai No.8’, ‘Lihongbao’, ‘Zaoheibao’, ‘Crimson Seedless’, ‘Balado Black’. EC. Epicarp (peel); MC. Mesocarp (pulp). **(a)** scale is 10 mm; **(b)** scale is 100 μm.

During fruit development (**Figure 3a**), the pericarp cell volume of ‘Thompson Seedless’, ‘Summer Black’, ‘Qiuhongbao’, ‘Wanheibao’, ‘Hutai No.8’, ‘Lihongbao’, ‘Zaoheibao’, and ‘Crimson Seedlees’ gradually increased. Their transitioned from a densely packed and intact morphology to a looser arrangement (**Figure 3b**). The pericarp thickness of ‘Summer Black’, ‘Hutai No.8’, ‘Lihongbao’, ‘Zaoheibao’ and ‘Crimson Seedless’ gradually decreased from the expansion stage to the ripening stage. At the expansion stage, pericarp cells of ‘Flame Seedless’, ‘Thompson White’, ‘Qiuhongbao’, ‘Jinghongbao’ and ‘Black Balado’ were loosely arranged. As fruit development progressed, the connections between adjacent cells weakened, and pulp cell separation increased. Meanwhile, the flattened and neatly arranged pericarp cells observed in ‘Wanheibao’, ‘Hutai No.8’, ‘Lihongbao’, ‘Zaoheibao’ and ‘Crimson Seedless’ may be associated with their more robust cell walls.

### 3.3 Changes in cell wall polysaccharide content during fruit development of different grape varieties

**Figure 4:**
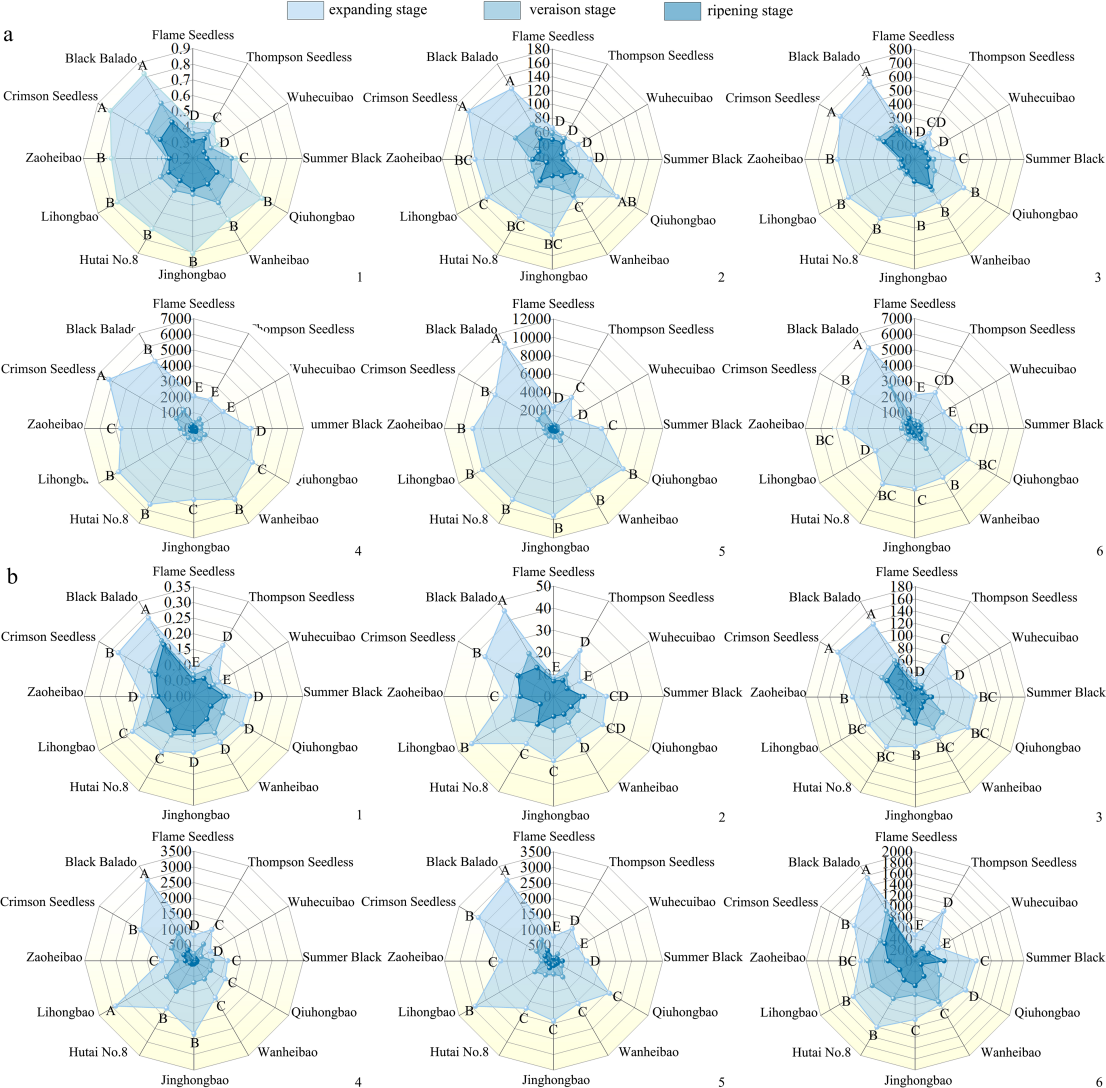
Changes in the contents of cell wall polysaccharides during the development of different varieties of grapes. **(a)**: Changes in the polysaccharide content of the pericarp cell walls during the development of different varieties of grapes; **(b)**: Changes in the polysaccharide content of pulp cell walls during the development of different varieties of grapes. Different capital letters in the figure indicate significant differences between different varieties (P ≤ 0.05). 1, 2, 3, 4, 5, 6 represent: cell wall material (mg·mg^-1^ FW), cellulose (mg·g^-1^ FW), hemicellulose (mg·g^-1^ FW), protopectin (μg·g^-1^ FW), chelator-soluble pectin (μg·g^-1^ FW), water soluble pectin (μg·g^-1^ FW) content.

During fruit development, the cell wall polysaccharide content in the 12 grape varieties showed a decreasing trend (**Figures 4a, b**). Notably, the decline of cell wall material content in the pulp (30.3%~64.8%) was more pronounced than that in the skin (23.9%~51.4%). The reduction in cell wall material content in the pericarp was greater from the expansion stage to the veraison stage (5.5%~49.5%) than from the veraison stage to the ripening stage (7.2%~47.8%). The contents of protopectin and chelator-soluble pectin showed the most rapid decline. Among the 12 varieties, the chelator-soluble pectin content decreased by 87.8%~97.7% in the skin, and by 73.7%~94.6% in the pulp. The reduction in water-soluble pectin content was greater in the skin (84.3%~92.5%) than in the pulp (43.1%~87.8%).

### 3.4 Changes in the activities of enzymes related to cell wall polysaccharide degradation during the development of grape berries of different varieties

**Figure 5:**
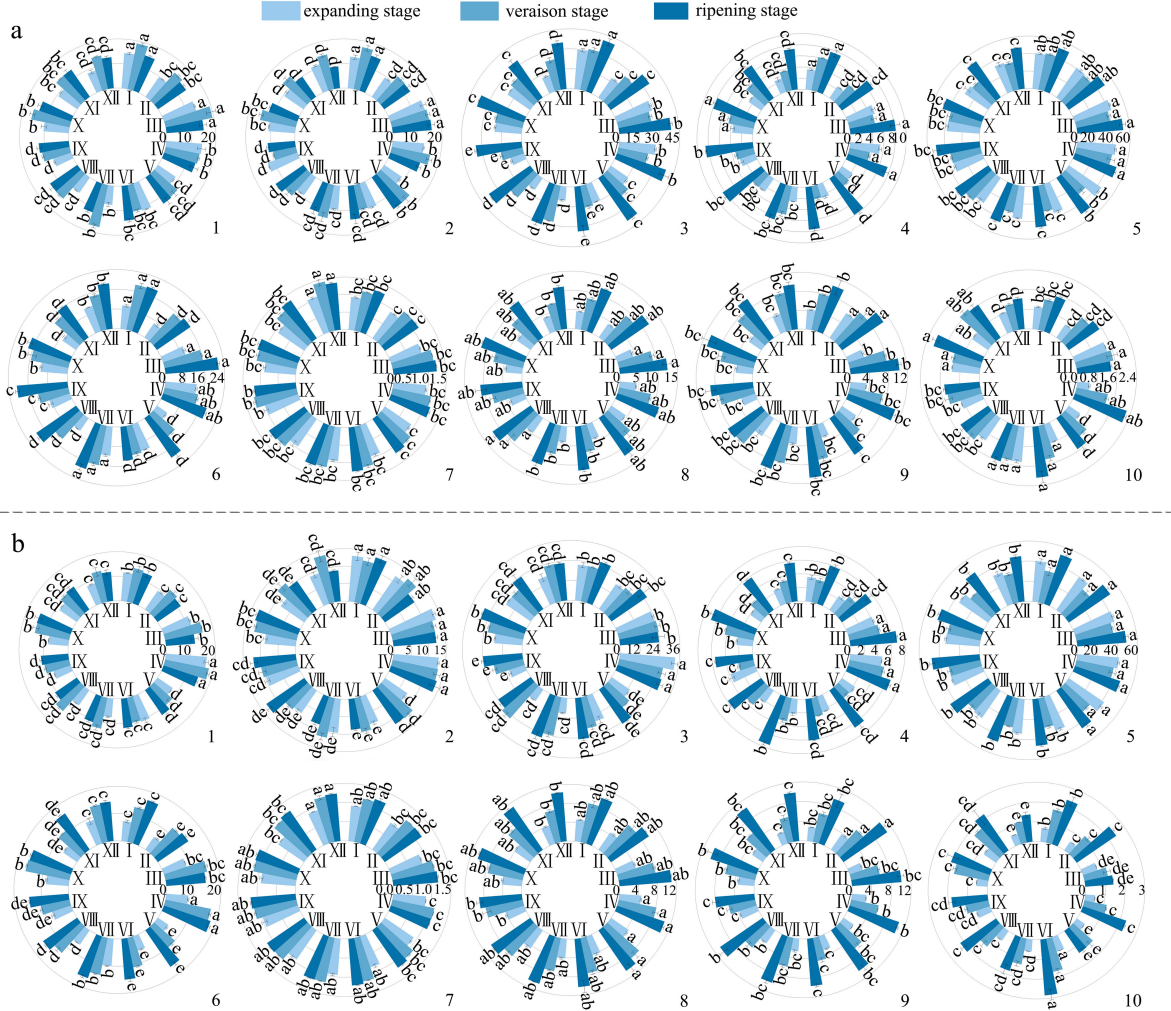
Changes in the activity of polysaccharide-degrading enzymes during the development of different varieties of grapes. **(a)**: Changes in the activity of degrading enzymes in the pericarp during the development of different varieties of grapes; **(b)**: Changes in the activity of degrading enzymes in the pulp during the development of different varieties of grapes. Different lowercase letters in the figure indicate significant differences between different varieties (P ≤ 0.05). I, II, III, IV, V, VI, VII, VIII, IX, X, XI, XII represent: ‘Flame Seedless’, ‘Thompson Seedless’, ‘Wuhecuibao’, ‘Summer Black’, ‘Qiuhongbao’, ‘Wanheibao’, ‘Jinghongbao’, ‘Hutai No.8’, ‘Lihongbao’, ‘Zaoheibao’, ‘Crimson Seedless’, ‘Balado Black’. 1, 2, 3, 4, 5, 6, 7, 8, 9, 10 represent: cellulase (μmol·min^-1^·mL^-1^), β-glucosidase (μmol·min^-1^·mL^-1^), xylanase (μmol·min^-1^·mL^-1^), xyloglucan endotransglycosylase (μmol·min^-1^·g^-1^ FW), β-mannanase (μmol·min^-1^·mL^-1^), polygalacturonase (μmol·min^-1^·mL^-1^), pectate lyase (μmol·min^-1^·mL^-1^), pectin methyl esterase (mmol·h^-1^·g^-1^ FW), β-galactosidase (μmol·min^-1^·mL^-1^), α-L-arabinofuranosidase (μmol·min^-1^·mL^-1^) activities.

During fruit development, the activities of xylanase, xyloglucan endotransglycosylase, β-mannanase, polygalacturonase, pectate lyase, pectin methylesterase, β-galactosidase, α-L-arabinofuranosidase in the skin and pulp of different varieties exhibited an overall increasing trend (**Figures 5a, b**). Among them, cellulase and β-glucosidase activities showed a smoother change, with an initial increase followed by a decrease. The activities of xylanase, xyloglucan endotransglycosylase and polygalacturonase increased more significantly. From the expansion to the ripening stage, xylanase activity increased by 0.37-2.55 times in the skin and 0.01-1.84 times in the pulp; xyloglucan endotransglycosylase activity by 0.38~2.37 times in the pericarp and 0.42~2.33 times in the pulp; and polygalacturonase activity increased 0.21-2.85 times in the skin and 0.58-2.43 times in the pulp. Pectate lyase activity increased rapidly by 16%-43% before the veraison and more slowly 1%-11% after the veraison. Pectin methylesterase and β-galactosidase exhibited higher activity increases during both the expansion-veraison and veraison-ripening stages. In grape skin, the activities of pectin methylesterase and β-galactosidase increased by 0.69-1.07 and 0.21-0.55 times, respectively, and in grape pulp by 0.29-1.53 and 0.05-1.02 times, respectively. Compared with the increase in the skin (1%~341%), α-L-arabinofuranosidase activity showed a more substantial increase in the pulp (85%~365%). The increased activities of these enzymes related to cell wall polysaccharide degradation likely facilitated polysaccharide breakdown, contributing to the development of easy-peeling traits in grapes.

### 3.5 Correlation analysis between skin-pulp adherence and cell wall polysaccharides in different varieties of grape berries

**Figure 6:**
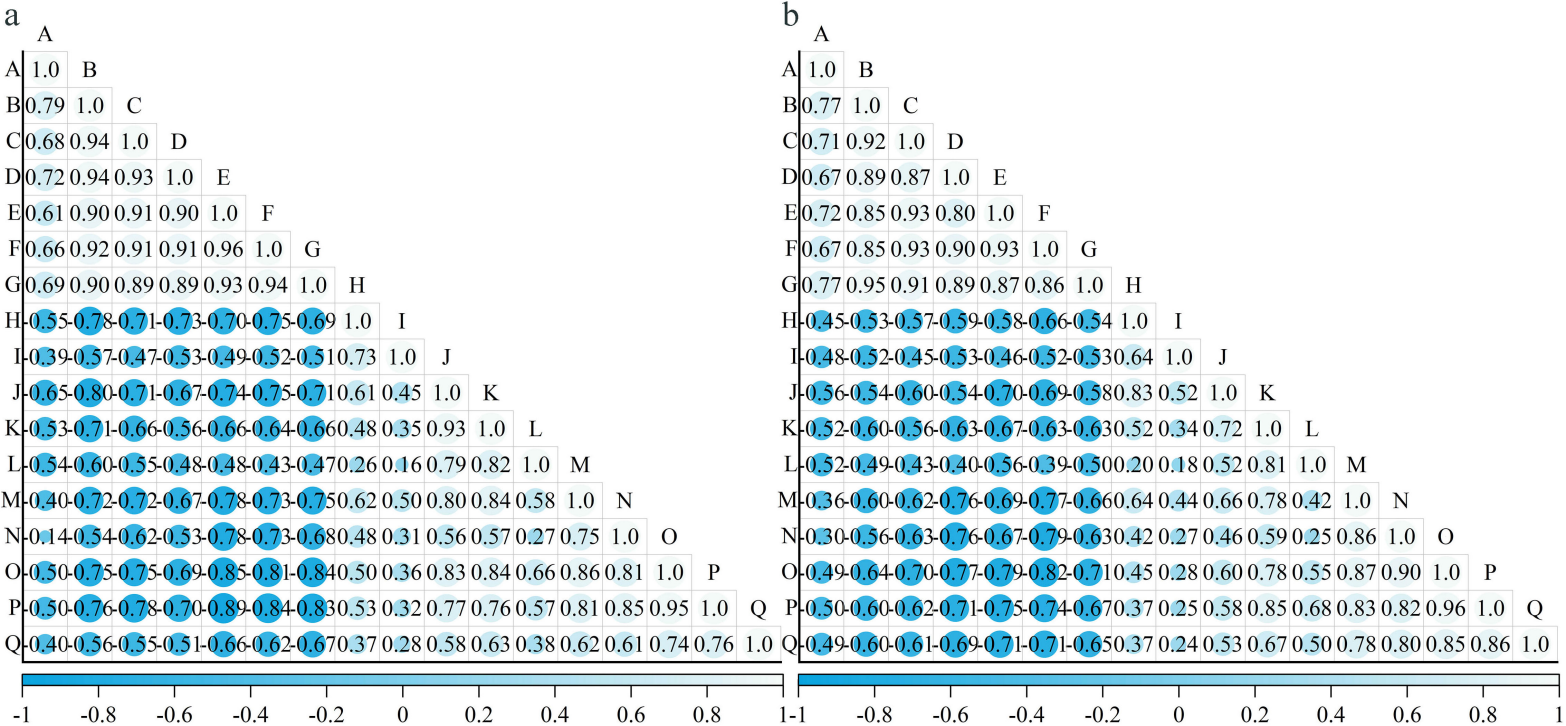
The correlation between polysaccharide-related indexes in different varieties of grape berries. **(a)**: Correlation between indicators related to the cell wall polysaccharides in the pericarp of different varieties of grapes; **(b)**: Correlation between indicators related to the cell wall polysaccharides in the pulp of different varieties of grapes. A: skin-pulp adherence, B: cell wall material, C: cellulose, D: hemicellulose, E: protopectin, F: chelator-soluble pectin, G: water soluble pectin, H: cellulase, I: β-glucosidase, J: xylanase, K: xyloglucan endotransglycosylase, L: β-mannanase, M: polygalacturonase, N: pectate lyase, O: pectin methyl esterase, P: β-galactosidase, Q: α-L-arabinofuranosidase.

Pearson correlation analysis was conducted between skin–pulp adherence and 16 polysaccharide-related indices. The results indicated that skin–pulp adherence was positively correlated with the content of cell wall polysaccharides in both skin and pulp, and negatively correlated with the activities of enzymes involved in their degradation (**Table 1**). Specifically, skin–pulp adherence was highly positively correlated with total cell wall material, cellulose, hemicellulose, protopectin, chelator-soluble pectin, and water-soluble pectin in the skin. It showed significant negative correlations with cellulase, xylanase, xyloglucan endotransglycosylase, β-mannanase, pectin methylesterase, and β-galactosidase, as well as with β-glucosidase, polygalacturonase, and α-L-arabinofuranosidase. However, the correlation with pectin lyase was not significant (**Table 1**). These findings suggest that low cell wall polysaccharide content in the skin and pulp, combined with high enzymatic activities related to polysaccharide degradation, may contribute to reduced skin–pulp adherence. Moreover, a significant negative correlation was observed between the content of cell wall polysaccharides and the activity of related degrading enzymes indicating that the degradation of cell wall polysaccharides is likely promoted by increased enzymatic activity; cellulase activity showed positive correlations with β-glucosidase, xylanase, xyloglucan endoglycosyltransferase, polygalacturonase, pectin lyase, pectin methylesterase, β-galactosidase, and α-L-arabinofuranosidase, but not with β-mannanase suggesting a potential synergistic relationship between cellulase and these enzymes in facilitating the development of the easy-peeling trait in grapes (**Figure 6**).

Table 1Correlation between peelability and cell wall polysaccharide related indexes in different varieties of grape berries.

**Table:** 

Part	Peelability	Cell wall material	Cellulose	Hemicellulose	Protopectin	chelator-soluble pectin	Water soluble pectin	Cellulase	β-glucosidase	Xylanase
Skin	Adherence	0.790^**^	0.683^**^	0.720^**^	0.608^**^	0.660^**^	0.693^**^	-0.552^**^	-0.393^*^	-0.645^**^
Pulp	Adherence	0.769^**^	0.713^**^	0.670^**^	0.722^**^	0.667^**^	0.775^**^	-0.449^**^	-0.479^**^	-0.560^**^

**Table:** 

Part	Peelability	Xyloglucan endotransglycosylase		β-mannanase	Polygalacturonase	Pectate lyase	Pectin methyl esterase	β-galactosidase	α-L-Arabinofuranosidase	
Skin	Adherence	-0.529^**^		-0.539^**^	-0.403^*^	-0.142	-0.501^**^	-0.498^**^	-0.398^*^	
Pulp	Adherence	-0.516^**^		-0.524^**^	-0.363^*^	-0.296	-0.493^**^	-0.498^**^	-0.490^**^	

**P<0.01, *P<0.05.

In this study, the cell wall polysaccharide content in the pulp was lower than that in the skin. However, the absolute difference between the correlation coefficients of skin- and pulp-dreived polysaccharide content with skin-pulp adherence was small. This suggests that the decline in skin-pulp adherence may be more influenced by the activity of cell wall polysaccharide degradation related enzymes and other factors that alter polysaccharide levels. Although the content of cell wall polysaccharides varied between the skin and pulp, a universally strong feedback regulation of polysaccharide content on the activity of enzymes related to cellular polysaccharide degradation may not exist.

### 3.6 Based least squares regression analysis of skin-pulp adherence and cell wall polysaccharides in different varieties of grape berries

**Figure 7:**
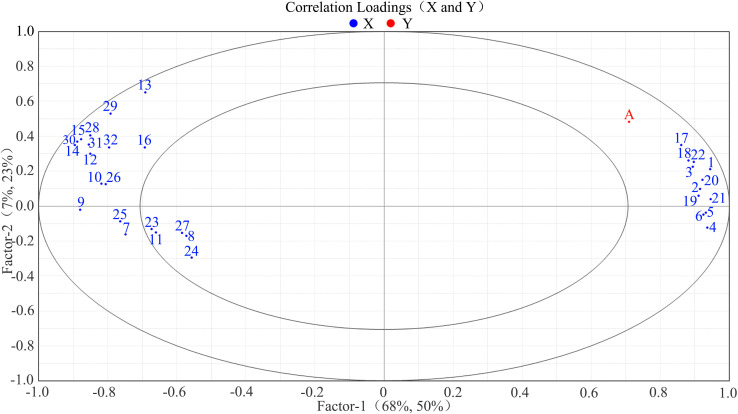
Correlation load of skin-pulp adherence with 32 cell wall polysaccharide-related indicators. Correlation load between skin-pulp adherence and 32 cell wall polysaccharide related indicators, the red color in the graph indicates the skin-pulp adherence, and the blue color indicates the cell wall polysaccharide indexes, and the skin-pulp adherence and cell wall polysaccharide indexes in the same region are more highly correlated the farther they are from the origin and the closer they are to each other; A: skin-pulp adherence; 1: peel cell wall material; 2: peel cellulose; 3: peel hemicellulose; 4: peel protopectin; 5: peel chelator-soluble pectin; 6: peel water-soluble pectin; 7: peel cellulase; 8: peel β-glucosidase; 9: peel xylanase; 10: peel xyloglucan. endotransglycosylase; 11: peel β-mannanase; 12: peel polygalacturonase; 13: peel pectate lyase; 14: peel pectin methyl esterase; 15: peel β-galactosidase; 16: peel α-L-arabinofuranosidase; 17: pulp cell wall material; 18: pulp cellulose; 19: pulp hemicellulose; 20: pulp protopectin; 21: pulp chelator-soluble pectin; 22: pulp water-soluble pectin; 23: pulp cellulase; 24: pulp β-glucosidase; 25: pulp xylanase; 26: pulp xyloglucan endotransglycosylase; 27: pulp β-mannanase; 28: pulp polygalacturonase; 29: pulp pectate lyase; 30: pulp pectin methyl esterase; 31: pulp β-galactosidase; 32: pulp α-L-arabinofuranosidase.

In the correlation loading plot, the shortest linear distance among the 32 indicators was observed between “17” (pulp cell wall material) and “A” (skin-pulp adherence). This suggests that a reduction in pulp cell wall material may be closely associated with decreased skin-pulp adherence (**Figure 7**). In contrast, “4” (skin protopectin), “5” (skin chelator-soluble pectin), and “6” (skin water-soluble pectin) were located farthest from “A” in the first and fourth quadrants, suggesting that changes in skin pectin content may exert less influence on skin-pulp adherence.

**Figure 8:**
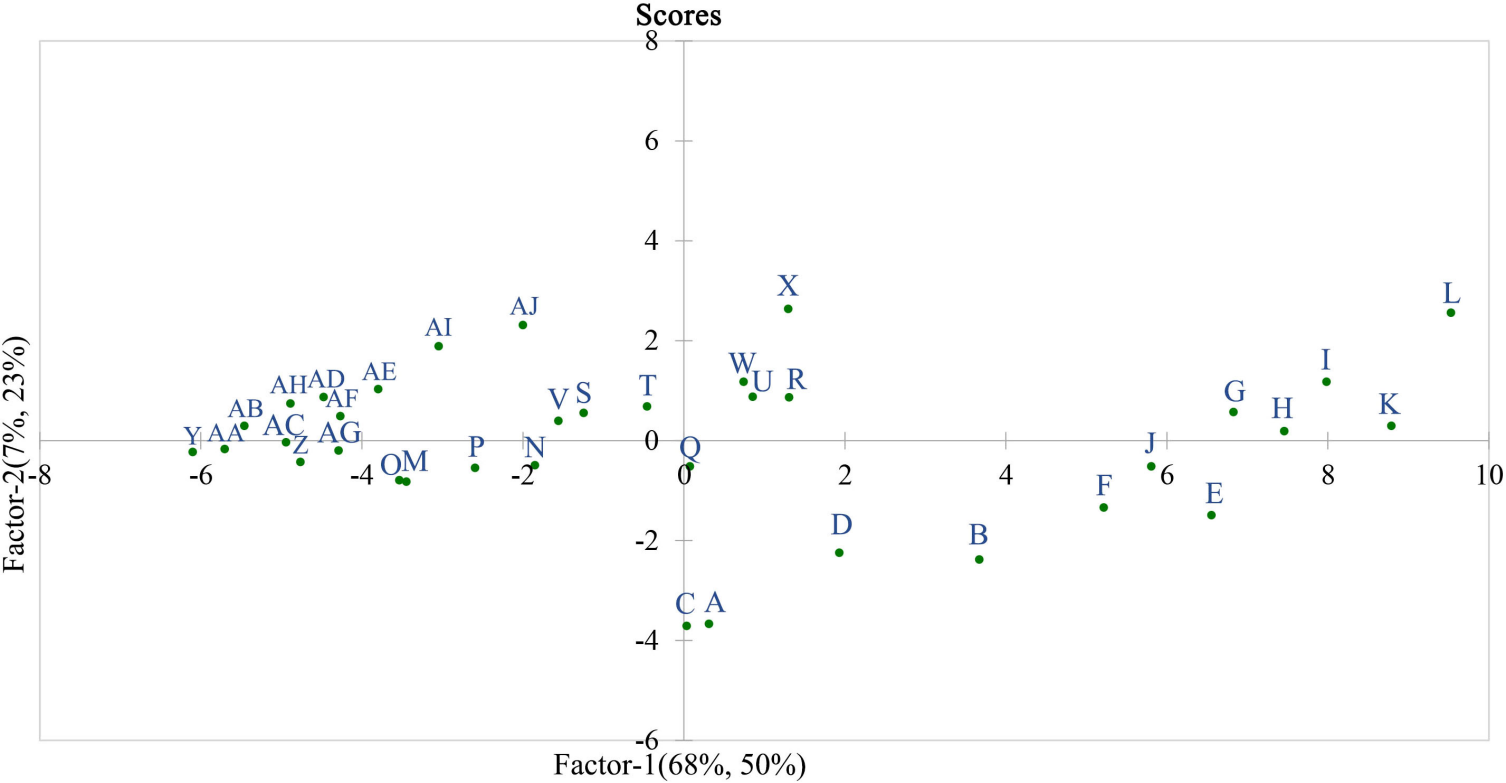
Sample observation score in partial least squares regression. A: ‘Flame Seedless’-expanding stage; B: ‘Thompson Seedless’-expanding stage; C: ‘Wuhecuibao’-expanding stage; D: ‘Summer Black’-expanding stage; E: ‘Qiuhongbao’-expanding stage; F: ‘Wanheibao’-expanding stage; G: ‘Jinghongbao’-expanding stage; H: ‘Hutai No.8’-expanding stage; I: ‘Lihongbao’-expanding stage; J: ‘Zaoheibao’-expanding stage; K: ‘Crimson Seedless’-expanding stage; L: ‘Black Balado’-expanding stage; M: ‘Flame Seedless’-veraison stage; N: ‘Thompson Seedless’-veraison stage; O: ‘Wuhecuibao’-veraison stage; P: ‘Summer Black’-veraison stage; Q: ‘Qiuhongbao’-veraison stage; R: ‘Wanheibao’-veraison stage; S: ‘Jinghongbao’-veraison stage; T: ‘Hutai No.8’-veraison stage; U: ‘Lihongbao’-veraison stage; V: ‘Zaoheibao’-veraison stage; W: ‘Crimson Seedless’-veraison stage; X: ‘Black Balado’-veraison stage; Y: ‘Flame Seedless’-ripening stage; Z: ‘Thompson Seedless’-ripening stage; AA: ‘Wuhecuibao’-ripening stage; AB: ‘Summer Black’-ripening stage; AC: ‘Qiuhongbao’-ripening stage; AD: ‘Wanheibao’-ripening stage; AE: ‘Jinghongbao’-ripening stage; AF: ‘Hutai No.8’-ripening stage; AG: ‘Lihongbao’-ripening stage; AH: ‘Zaoheibao’-ripening stage; AI: ‘Crimson Seedless’-ripening stage; AJ: ‘Black Balado’-ripening stage.

Based on the 32 measured indices related to cell wall polysaccharides, the skin-pulp adherence of different grape varieties at various stages was evaluated using principal component analysis (**Figure 8**). “L” (‘Black Balado’-expanding stage) and “K” (‘Crimson Seedless’-expanding stage) were located in the first quadrant and farthest from the origin, indicating that these two varieties exhibited the highest skin-pulp adherence during the expanding stage, consistent with the measured values. Using the lowest observed values on the horizontal and vertical axes as the origin, point “X” (‘Black Balado’-veraison stage) showed lower skin-pulp adherence than points “B” (‘Thompson Seedless’-expanding stage), “F” (‘Wanheibao’-expanding stage), “J” (‘Zaoheibao’-expanding stage), “E” (‘Qiuhongbao’-expanding stage), “G” (‘Jinghongbao’-expanding stage), “H” (‘Hutai No.8’-expanding stage), and “I” (‘Lihongbao’- expanding stage). This suggests that the decrease in skin-pulp adherence may be smaller than the decrease in cell wall polysaccharide content. In addition, cell wall polysaccharide content is a key factor influencing variation in skin-pulp adherence. However, other factors contributing to skin-pulp adherence remain to be further investigated. The variation in skin-pulp adherence among grape varieties may be closely linked to differences in total cell wall material or polysaccharide content during the expansion stage. This may reflect that varieties with higher adherence possess more active polysaccharide synthesis pathways and elevated activities of related enzymes.

**Figure 9:**
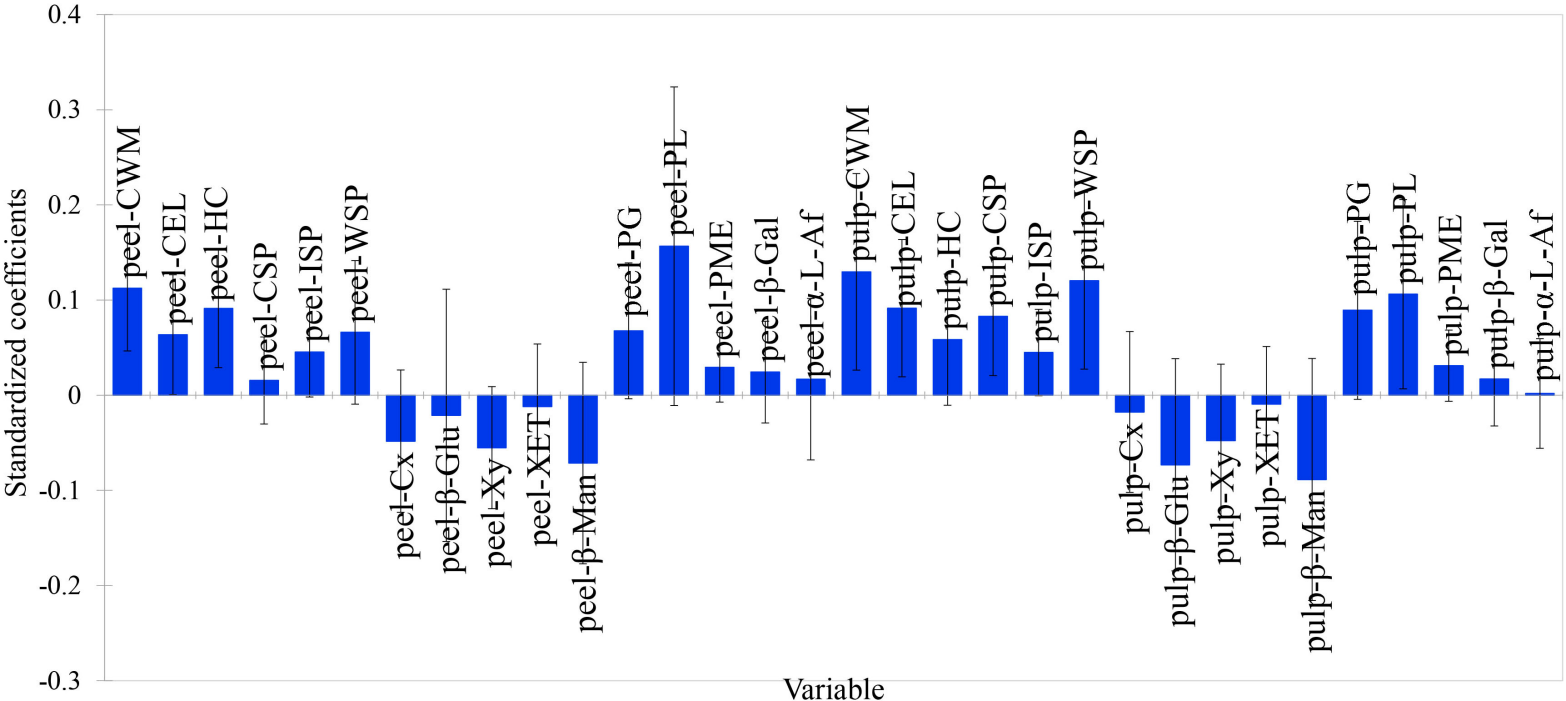
The standardized regression coefficients for 32 cell wall polysaccharide-related indicators. peel-CWM: peel cell wall material; peel-CEL: peel cellulose; peel-HC: peel hemicellulose; peel-CSP: peel protopectin; peel-ISP: peel chelator-soluble pectin; peel-WSP: peel water-soluble pectin; peel-Cx: peel cellulase; peel-β-Glu: peel β-glucosidase; peel-Xy: peel xylanase; peel-XET: peel xyloglucan endotransglycosylase; peel-β-Man: peel β-mannanase; peel-PG: peel polygalacturonase; peel-PL: peel pectate lyase; peel-PME: peel pectin methyl esterase; peel-β-Gal: peel β-galactosidase; peel-α-L-Af: peel α-L-arabinofuranosidase; pulp-CWM: pulp cell wall material; pulp-CEL: pulp cellulose; pulp-HC: pulp hemicellulose; pulp-CSP: pulp protopectin; pulp-ISP: pulp chelator-soluble pectin; pulp-WSP: pulp water-soluble pectin; pulp-Cx: pulp cellulase; pulp-β-Glu: pulp β-glucosidase; pulp-Xy: pulp xylanase; pulp-XET: pulp xyloglucan endotransglycosylase; pulp-β-Man: pulp β-mannanase; pulp-PG: pulp polygalacturonase; pulp-PL: pulp pectate lyase; pulp-PME: pulp pectin methyl esterase; pulp-β-Gal: pulp β-galactosidase; pulp-α-L-Af: pulp α-L-arabinofuranosidase.

In both the skin and pulp, the standardized regression coefficients of cellulase, β-glucosidase, xylanase, xyloglucan endoglycosyltransferase, and β-mannanase were negative, while those of polygalacturonase, pectin lyase, pectin methyl esterase, β-galactosidase, and α-L-arabinofuranosidase were positive. However, polygalacturonase, pectin lyase, pectin methyl esterase, β-galactosidase, and α-L-arabinofuranosidase exhibited negative correlation coefficients with skin-pulp adherence force (**Figure 9**). This suggests that the five enzymes involved in pectin degradation play a relatively minor role in regulating skin-pulp adherence compared to cellulase, β-glucosidase, xylanase, xyloglucan endoglycosyltransferase, and β-mannanase. Since the linear distances between pulp cellulose, pulp cell wall cell wall material and skin-pulp adherence were the shortest in the correlation loadings plot, the absolute values of the standardized regression coefficient for pulp cellulase (-0.018) was 37.5% of that for pulp xylanase (-0.048) and 20.2% of that for pulp β-mannanase (-0.089). respectively. It suggests that the degradation of hemicellulose, which is involved in cellulose cross-linking, may be one of the factors constituting the decrease in the cell wall material content of cellulose and cell wall.

### 3.7 Principal component analysis of cell wall polysaccharide related indexes of different varieties of grape berries

Principal component scores of 12 grape varieties at different ripening stages were predicted using cell wall polysaccharide content as negative indicators and enzymes related to cellular polysaccharide degradation activities as positive indicators (**Table 2**). At ripening stage, the peelability ranking was as follows: ‘Flame eedless’, ‘Thompson Seedless’, ‘Wuhecuibao’, ‘Qiuhongbao’, ‘Summer Black’, ‘Lihongbao’ ‘Hutai No.8’ ‘Jinghongbao’, ‘Zaoheibao’ ‘Wanheibao’, ‘Crimson Seedless’, ‘Black Balado’. During the expansion stage, the varieties were ranked as follows: ‘Wuhecuibao’, ‘Flame Seedless’, ‘Thompson Seedless’, ‘Summer Black’, ‘Wanheibao’, ‘Qiuhongbao’, ‘Zaoheibao’, ‘Jinghongbao’, ‘Hutai No.8’, ‘Crimson Seedless’, ‘Lihongbao’, and ‘Black Balado’. ‘Qiuhongbao’, ‘Lihongbao’, and ‘Hutai No.8’ showed lower scores during the expansion stage and higher scores at maturity, while ‘Wanheibao’, ‘Zaoheibao’, and ‘Wuhecuibao’ exhibited the opposite trend (**Table 3**). This suggests that these six varieties underwent more substantial changes in cell wall polysaccharide content and cell wall polysaccharide degradation related enzymes activity during development, resulting in significant variation in peelability.

**Table 2:** Load values and contribution rates of cell wall polysaccharide related indicators.

Indicator	Principal component 1	Principal component 2	Principal component 3	Principal component 4
Peel cell wall material	-0.931	0.195	-0.150	0.108
Pulp cell wall material	-0.850	0.186	-0.140	0.345
Peel cellulose	-0.905	0.172	-0.051	0.091
Pulp cellulose	-0.880	0.182	-0.013	0.333
peel hemicellulose	-0.888	0.286	-0.016	0.187
pulp hemicellulose	-0.917	0.149	0.125	0.180
peel protopectin	-0.933	0.134	0.160	0.049
pulp protopectin	-0.904.’	0.076	-0.126	0.235
peel chelator-soluble pectin	-0.930	0.198	0.132	0.044
pulp chelator–soluble pectin	-0.936	0.216	0.067	0.158
peel water-soluble pectin	-0.913	0.152	0.126	0.152
pulp water-soluble pectin	-0.887	0.152	-0.111	0.272
peel cellulase	0.736	-0.517	0.062	0.160
pulp cellulase	0.666	-0.434	0.190	0.381
peel β-glucosidase	0.561	-0.522	0.124	0.215
pulp β-glucosidase	0.515	-0.520	0.288	0.266
peel xylanase	0.865	0.207	0.310	0.103
pulp xylanase	0.747	-0.117	0.356	0.340
Peel xyloglucan endotransglycosylase	0.828	0.384	0.238	0.116
pulp xyloglucan endotransglycosylase	0.815	0.377	0.261	0.194
peel β-mannanase	0.647	0.458	0.541	-0.083
pulp β-mannanase	0.574	0.565	0.544	-0.095
peel polygalacturonase	0.879	0.113	-0.019	0.217
pulp polygalacturonase	0.890	0.069	-0.172	0.290
peel pectate lyase	0.740	0.146	-0.502	0.293
pulp pectate lyase	0.837	0.066	-0.468	0.085
peel pectin methyl esterase	0.925	0.305	-0.144	0.021
pulp pectin methyl esterase	0.922	0.253	-0.207	0.013
peel β-galactosidase	0.913	0.227	-0.235	0.032
pulp β-galactosidase	0.885	0.413	-0.092	0.034
peel-α-L-arabinofuranosidase	0.722	0.217	-0.299	-0.021
pulp-α-L-arabinofuranosidase	0.812	0.279	-0.253	-0.004
eigenvalue	22.151	2.816	2.110	1.207
variance explained rate (%)	69.222	8.801	6.276	3.773
Cumulative contribution (%)	69.222	78.023	84.299	88.072

**Table 3:** Comprehensive score and ranking of peelability in different varieties at different stages.

Sample	1st Principal Component Score	2nd Principal Component Score	3rd Principal Component Score	4th Principal Component Score	Combined Score	Rank
‘Flame Seedless’-ripening stage	-626.88	220.24	134.72	166.78	-453.954	1
‘Thompson Seedless’-ripening stage	-907.37	270.25	119.20	212.79	-668.556	2
‘Wuhecuibao’-ripening stage	-975.43	283.41	136.22	234.76	-718.576	3
‘Qiuhongbao’-ripening stage	-1154.53	313.31	180.34	227.18	-853.539	4
‘Summer Black’-ripening stage	-1311.84	347.48	102.10	333.30	-974.793	5
‘Lihongbao’-ripening stage	-1411.70	346.58	164.95	293.90	-1050.58	6
‘Hutai No.8’-ripening stage	-1411.60	353.48	133.74	312.55	-1051.23	7
‘Jinghongbao’-ripening stage	-1422.74	356.67	127.81	331.48	-1059.29	8
‘Flame Seedless’-veraison stage	-1641.52	353.79	203.52	283.05	-1228.21	9
‘Zaoheibao’-ripening stage	-1654.72	387.27	114.72	378.52	-1237.47	10
‘Wanheibao’-ripening stage	-1754.59	424.46	204.91	347.42	-1307.16	11
‘Wuhecuibao’-veraison stage	-1891.12	386.31	211.01	324.30	-1418.83	12
‘Summber Black’-veraison stage	-2407.47	467.03	147.87	472.30	-1814.77	13
‘Crimson Seedless’-ripening stage	-2481.59	546.31	142.40	525.02	-1863.23	14
‘Thompson Seedless’-veraison stage	-2501.59	466.19	176.16	452.65	-1887.65	15
‘Black Balado’-ripening stage	-2898.88	615.57	134.35	624.82	-2180.58	16
‘Jinghongbao’-veraison stage	-3718.05	696.23	234.09	643.14	-2808.47	17
‘Qiuhongbao’-veraison stage	-3847.00	715.54	297.33	613.62	-2904.66	18
‘Zaoheibao’-veraison stage	-4185.63	779.89	292.50	710.05	-3160.59	19
‘Hutai No.8’-veraison stage	-4476.15	799.40	188.01	823.84	-3389.56	20
‘Lihongbao’-veraison stage	-4500.13	819.42	174.23	824.44	-3407.36	21
‘Wanheibao’-veraison stage	-5596.48	1051.37	412.34	899.79	-4225.69	22
‘Crimson Seedless’-veraison stage	-6009.33	1131.62	468.44	871.86	-4539.35	23
‘Wuhecuibao’-expanding stage	-7899.32	1456.23	900.92	1012.76	-5955.54	24
‘Flame Seedless’-expanding stage	-8088.56	1473.13	880.36	1041.36	-6102.83	25
‘Black Balado’-veraison stage	-8882.63	1607.53	744.70	1388.00	-6708.33	26
‘Thompson Seedless’-expanding stage	-11326.45	2089.33	1066.20	1509.87	-8552.84	27
‘Summer Black’-expanding stage	-14167.68	2598.92	1529.32	1697.98	-10694	28
‘Wanheibao’-expanding stage	-19231.09	3522.42	2188.25	2079.96	-14518.1	29
‘Qiuhongbao’-expanding stage	-20186.66	3815.81	2272.77	2212.44	-15228.1	30
‘Zaoheibao’-expanding stage	-20641.20	3884.54	2390.34	2243.01	-15568.8	31
‘Jinghongbao’-expanding stage	-21897.35	4019.64	2218.77	2495.16	-16544	32
‘Hutai No.8’-expanding stage	-22284.69	4081.51	2369.34	2501.53	-16831.3	33
‘Crimson Seedless’-expanding stage	-23143.37	4206.41	2394.78	2766.07	-17480.6	34
‘Lihongbao’-expanding stage	-23229.92	4231.29	2151.15	2758.28	-17563.8	35
‘Black Balado’-expanding stage	-27843.75	5106.01	2632.35	3463.49	-21038.2	36

## 4 Discussion

Peelability is a key indicator of the quality of horticultural products (Xu et al., 2023). In general, fruit becomes softer and easier to peel as it ripens (Tao et al., 2024). Skin-pulp adherence is a visual and measurable indicator of fruit peelability. In this study, comparison of skin-pulp adherence among different grape varieties revealed a general decline in adhesion during development. In addition, significant differences in adhesion were observed between varieties. Cluster analysis showed that ten Eurasian grape varieties could be grouped into two categories: ‘Black Balado’ was classified as a difficult-to-peel variety, while the remaining nine were considered easy-to-peel. This indicates that peelability is strongly influenced by varietal differences. Similar results have been reported in mandarin varieties (Yu et al., 2021). The anatomical structure of the pericarp also affects peelability (Minamikawa et al., 2022). During the expansion stage of development, peel cells in the twelve grape varieties changed from compact to loosened structures, Varieties such as ‘Summer Black’, ‘Qiuhongbao’, ‘Wanheibao’, ‘Jinghongbao’, ‘Hutai No.8’, ‘Lihongbao’, and ‘Zaoheibao’ exhibited more loosely arranged pericarp cells compared to ‘Flame Seedless’, ‘Thompson Seedless’, ‘Wuhecuibao’, and ‘Black Balado’, which showed higher skin-pulp adherence. The peel thickness of ‘Summer Black’, ‘Hutai No.8’, ‘Lihongbao’, ‘Zaoheibao’, and ‘Crimson Seedless’ was lower at maturity compared to the swelling stage, suggesting that both peel looseness and thickness are correlated with ease of peeling. This is consistent with the findings of Gu et al. (2022) and Jia et al. (2022). Yu et al. (2021) reported that pericarp thickness was not associated with peelability in mandarin species, possibly due to interspecific differences. In this study, no distinct separation zone was observed between the pericarp and pulp, suggesting that the ease of peeling may be closely related to reduced structural integrity of the cell wall (Tee et al., 2011).

Polysaccharides are major components of fruit cell walls and hold significant application value (Ren et al., 2020). During fruit development, pectic polysaccharides undergo a gradual transformation from insoluble forms to soluble pectin and pectic acid, leading to a reduction in intercellular adhesion (Li et al., 2024). In this study, significant differences were observed in the contents of cell wall polysaccharides-including total cell wall material, cellulose, hemicellulose, and pectin-across different grape varieties and between peel and pulp tissues. These components consistently declined during fruit development, suggesting that cell wall polysaccharides in both the peel and pulp may jointly influence skin-pulp adherence. In citrus fruits, the content of cell wall polysaccharides in the peel gradually decreases during ripening, while the activity of polysaccharide degradation related enzymes and the expression levels of their corresponding genes continue to rise. Moreover, easy-to-peel citrus varieties generally contain lower levels of hemicellulose, cellulose, and pectin compared to hard-to-peel varieties (Zhang et al., 2021). Similar findings have been reported in *Actinidia eriantha* (Tao et al., 2024) and *Lycium barbarum* (Hu et al., 2023), where changes in cell wall polysaccharide content were shown to affect the development of peelability traits.

In our study, a highly significant positive correlation was observed between skin-pulp adherence force and the content of cell wall polysaccharides in the peel, which is consistent with the findings of Zhang et al. (2021), suggesting that alterations in peel cell wall composition influence skin-pulp adherence. Notably, a similarly strong positive correlation was also found between skin-pulp adherence and both the total cell wall material and polysaccharide content in the pulp. During fruit development, as skin-pulp adherence declined, the contents of cellulose, hemicellulose, and pectin in both the pulp and peel gradually decreased. This suggests that softening of both peel and pulp tissues may occur simultaneously, thereby reducing the mechanical resistance during separation. At the same developmental stage, ‘Flame Seedless’, ‘Thompson Seedless’, and ‘Wuhecuibao’ exhibited lower levels of cellulose, hemicellulose, and pectin in both peel and pulp compared to ‘Black Balado’, indicating that differences in cell wall polysaccharide content may affect the structural integrity of the cell wall and contribute to varietal differences in skin-pulp adherence. This observation is consistent with the findings of Li R, (2018), who reported lower pectin content in fruits with easier peel separation. Furthermore, our study demonstrated a general decreasing trend in skin-pulp adherence across grape varieties, which aligns with Zhang et al. (2021), where fruit at higher maturity stages tended to exhibit improved peelability.

Enzymes related to cell wall polysaccharide metabolism, such as pectinase, cellulase, and β-galactosidase, are known to play crucial roles in fruit ripening and softening, and are also involved in regulating fruit peelability (Zhang et al., 2021; Uluisik and Seymour, 2020). In this study, during the late stages of fruit development, the contents of total dry matter, cellulose, hemicellulose, and pectin in the cell wall gradually decreased. Meanwhile, the activities of a range of enzymes related to cellular polysaccharide degradation-including cellulase, β-glucosidase, xylanase, xyloglucan endotransglycosylase, β-mannanase, polygalacturonase, pectate lyase, pectin methylesterase, β-galactosidase, and α-L-arabinofuranosidase-significantly increased. The rapid elevation in enzyme activity suggests that these enzymes promote the degradation of cell wall polysaccharides, thereby facilitating fruit softening and peelability. In this study, the skin-pulp adherence showed a significant negative correlation with cellulase activity, while its correlation with pectin lyase was not significant. This differs from the findings of Zhang et al. (2021), indicating potential species-specific differences in the relationship between cell wall polysaccharide-enzymes related to cellular polysaccharide degradation activities and peel-pulp adherence. The contents of protopectin, chelator-soluble pectin, and water-soluble pectin were higher in the peel than in the pulp, and the reduction in pectin content during fruit development was also more pronounced in the peel. However, the correlation coefficients between peel–flesh adhesion and pectin content were lower in the peel than in the pulp. These findings suggest that pectin-degrading enzyme activity in the peel may not be the dominant factor influencing skin-pulp adherence. Prakash et al. (2017) reported that hemicellulose degradation had a greater impact on peelability. This is consistent with our results, which show that changes in peel hemicellulose content were closely associated with variations in skin-pulp adherence. Similarly, Yu et al. (2024) found that pectinase activity was higher in varieties with lower adherence. However, in our study, the standardized regression coefficients for peel cellulose and hemicellulose were higher than those for pectin components, indicating that reduced adhesion may primarily result from degradation of the structural components of the cell wall rather than pectin dissolution in the middle lamella. This contrasts with the findings of Liu et al. (2024), suggesting that the major polysaccharides influencing skin-pulp adherence may vary among fruit species. Notably, β-mannanase exhibited the highest negative standardized regression coefficient, indicating that it may be a key enzyme involved in regulating skin-pulp adherence. This aligns with Prakash et al. (2017), who reported elevated activities of xylanase and β-mannanase in varieties with easily separable peels. However, Jiao et al. (2018) argued that hemicellulase activity might not be directly associated with fruit texture. In our study, the decline in skin-pulp was less pronounced than the decrease in cell wall polysaccharide content, suggesting that varietal differences in polysaccharide levels during the fruit expansion stage may play a pivotal role in determining adhesion strength.

## 5 Conclusion

Lower skin–pulp adherence in grape berries was closely associated with reduced cell wall polysaccharide content and increased activity of enzymes related to cell wall degradation. Fruits at more advanced maturity stages were generally easier to peel. Loosening of the peel cell layer may serve as a morphological indicator of peelability. Partial least squares regression analysis revealed that changes in pulp cell wall composition were strongly correlated with variations in skin-pulp adherence. Among the cell wall polysaccharide components, the cellulose and cell wall material content in the pulp exhibited the strongest influence on skin–pulp adherence. In terms of degradation-related enzymes, cellulase, β-glucosidase, xylanase, xyloglucan endotransglycosylase, and β-mannanase were identified as key contributors. Based on cluster analysis of skin–pulp adherence during the ripening period, ten Eurasian grape varieties were classified into two distinct groups: easily peelable varieties, including ‘Flame Seedless’, ‘Thompson Seedless’, ‘Wuhecuibao’, ‘Zaoheibao’, ‘Wanheibao’, ‘Jinghongbao’, ‘Lihongbao’, ‘Qiuhongbao’, and ‘Crimson Seedless’; and the difficult-to-peel variety, ‘Black Balado’.

## Data Availability

The original contributions presented in the study are included in the article/supplementary material. Further inquiries can be directed to the corresponding authors.
